# Accident or abuse? Differential diagnosis of contact burns from radiators/heaters in children

**DOI:** 10.1007/s12024-024-00875-8

**Published:** 2024-08-13

**Authors:** A. Amadasi, C. Schönfeld, S. Etzold

**Affiliations:** 1 Gewaltschutzambulanz - Institute of Legal Medicine and Forensic Sciences, Charité- Universitätsmedizin Berlin, Turmstrasse 21, 10559 Berlin, Germany; 2 Gewaltschutzambulanz Bremen, Klinikum Bremen Mitte, Gesundheit Nord, Sankt- Jürgen-Straße 1, 28205 Bremen, Germany

**Keywords:** Forensic medicine, Contact burns, Children, Radiator, Heater, Accident, Child abuse, Scene investigation

## Abstract

Contact burns in children are not uncommon and are often due to accidental contact. Medico-legal assessment is of paramount importance in these contexts to identify cases of abuse. In three cases of burns caused by contact with radiators or a portable heater -two accidental and one deliberate- thorough medico-legal assessment, combined with on-site event reconstruction, enabled accurate diagnoses. Accidental burns displayed a ‘pattern’ compatible with the incandescent instrument but were more irregular, with different depths and in different parts of the body. In contrast, intentional burns were uniform in depth, distribution and localisation, inconsistent with accidental events. In this context, the on-site inspection and direct evaluation of the objects involved were crucial in the medico-legal assessment. These are indispensable elements for a thorough analysis and abuse recognition.

## Introduction

Burn injuries in children are a significant area of concern in pediatric healthcare due to their frequency and potential long-term physical and psychological effects [1–4]. In such a scenario, contact burns, a subtype of thermal burns, occur when the skin contacts a hot surface directly. These injuries are common in the pediatric population, due to children’s natural curiosity and their still-developing sense of danger, often leading to unintentional contact with hot objects. The pattern, location, and severity of burns, alongside the consistency (or lack thereof) with the caregiver’s account, are critical in differentiating accidental from non-accidental injuries [5–11].

Typical characteristics of contact burns include well-demarcated edges, uniform depth, blistering and tissue damage. Medical assessment is crucial to determine burn severity, potential complications, and appropriate treatment, also considering the fact that many take place in a domestic context [12–18].

Radiators, commonly used in homes for heating, can reach temperatures high enough to cause severe burns upon brief contact. In children, the most commonly affected areas are the hands and fingers, followed by the lower extremities, especially when toddlers or young children accidentally touch or fall against the radiator surface [19–22]. In this context, the possibility of child abuse must always be considered. The following three cases highlight the importance of medico-legal diagnosis and event reconstruction for accurate differential diagnosis between accidental and non-accidental events.

### Case 1

**Fig. 1:**
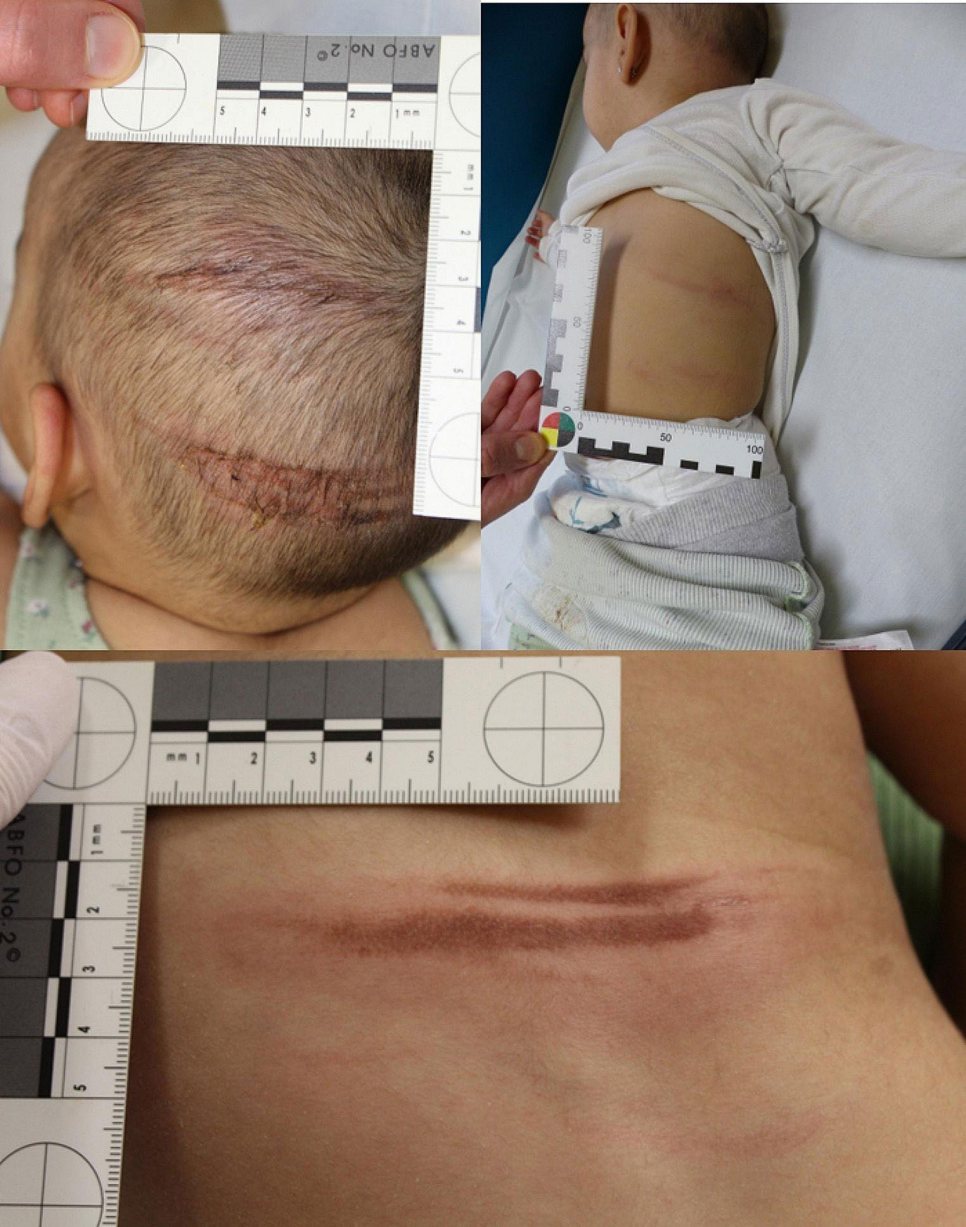
Case 1: first and second – degree burns on the head, left flank and back

A one-year-old girl was presented by her mother in the emergency room with burns on different parts of her body. At first, the mother revealed that she noticed the burns on the child’s body in the morning, stating that they were not present the previous day. At the examination, no major pathologies were reportedly found, overall the child was in good health. The child showed first and second degree burns on her head, the top of her left ear, the left side of her belly, her back, the fingertips of her right hand, her left arm, elbow and forearm. The burns on the head, flank and back appeared as parallel stripes, while the left arm had 1–2 degree burns with blisters (Fig. 1).

In order to obtain a possible reconstruction of the dynamic and an explanation for the burns, an inspection of the child’s home was carried out. First of all, the child’s cot was examined, which was in a corner of the room with a ribbed radiator under the window and next to the cot. It was ascertained that it would have been possible to reach the heater with the extremities (arms and legs), if the extremities were pushed through the cot´s bars, but this would have not explained the burns detected on the entire body.

**Fig. 2:**
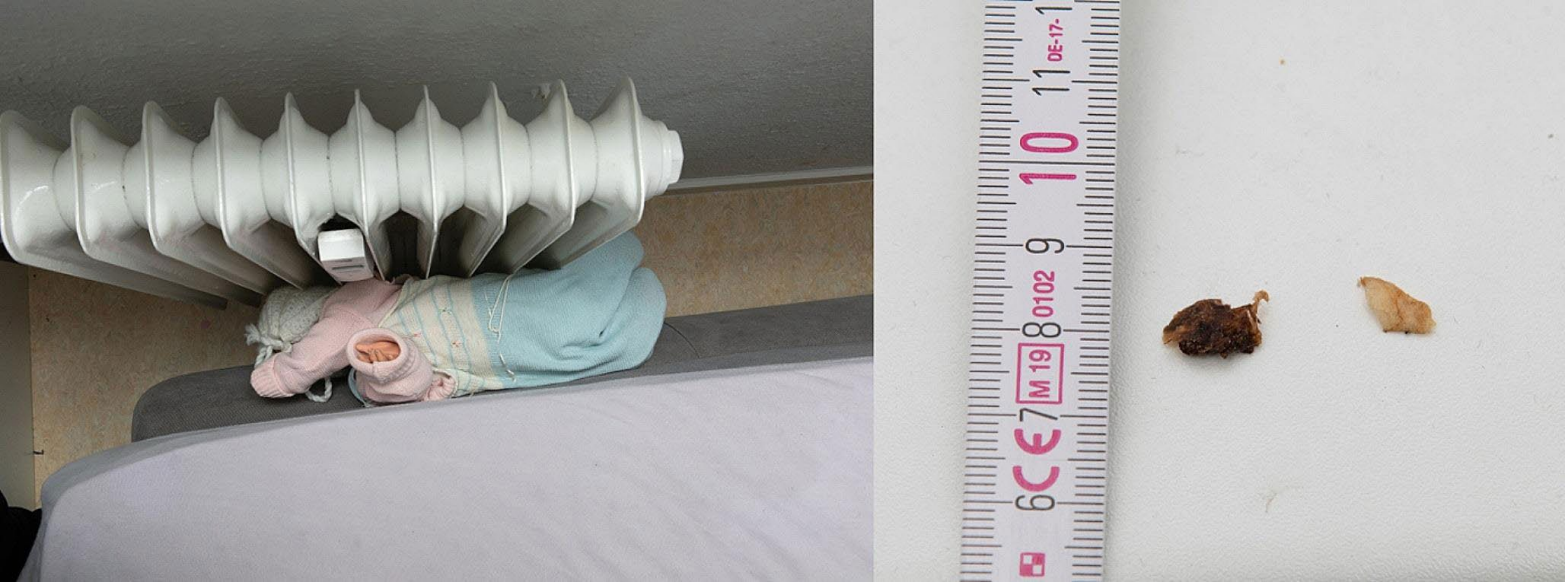
Case 1 - Reconstruction of the position of the body and contact points with the radiator (left). Hair and skin residues found on the radiator (right)

The investigation was then extended to the parents’ bedroom. There was a large bed with a thick mattress topper in the style of a box-spring bed; the mattress could be moved, but this requires a great deal of effort. The headboard of the bed stands against a wall. One side of the bed was also against the wall, on which there is a window and a ribbed radiator underneath. The side end of the bed was directly adjacent to the ribbed radiator. The top edge of the radiator was approximately 72 cm above floor level, height of the bed from floor level with mattress 59 cm, without mattress approx. 31 cm. On the radiator on the 5th (on the side facing the 6th rib) and 6th rib (on both sides) approximately 33 to 40 cm above floor level, tiny adhesions suspicious of skin residue and a short blackish hair were found. After examining the circumstances on site, it was conceivable that the little girl was actually lying in the parents´ bed and then fall between the mattress and the radiator, getting stuck and thus sustaining the burns. With a doll comparable in size to that of the child, a simulation of the positioning of the child in relation to the contact points of the body with the radiator was also carried out, demonstrating full compatibility with the burn patterns (Fig. 2).

The mother later admitted that she had put the child on the bed in the parents´ bedroom and then left the room. She had become aware of the child’s crying and had then found the child lying on the radiator. This also indicates a certain period of contact, of at least a few seconds, between the anatomical parts of the child’s body and the hot parts of the radiator, which led to the strip-like injuries consistent with the parts of the radiator, in addition to the injuries to the arm and hand probably caused by the child’s movements in an attempt to free herself. From a forensic point of view, this statement can be reconciled with the burn pattern, therefore ruling out an intentional contact.

### Case 2

**Fig. 3:**
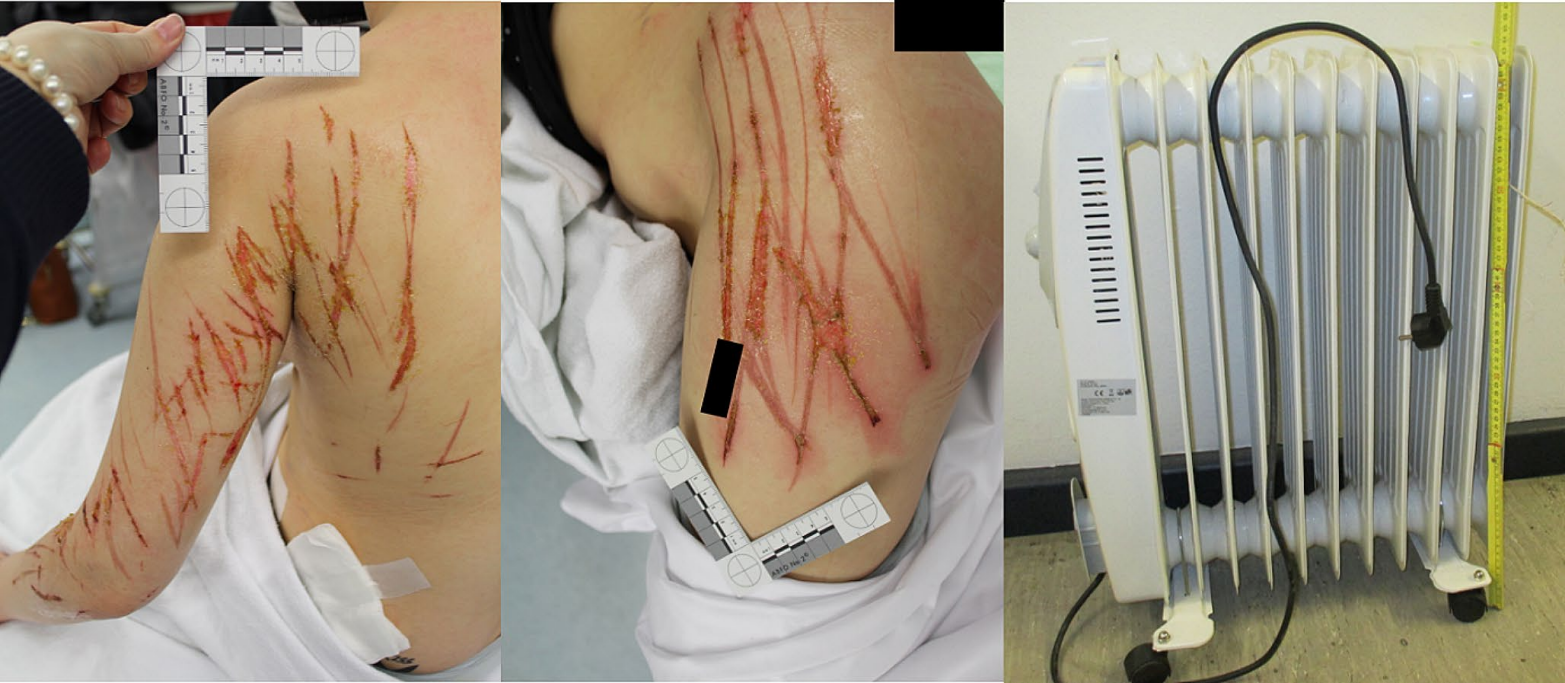
Case 2 - first and second – degree burns on the back and left arm (left) and on the outer part of the hip/left thigh (middle). The electrical radiator responsible for the injuries (right)

The case is that of a 12-year-old girl that was hospitalized with first and second degree burns on her left back, left arm and left leg. The burns were in the form of intersecting stripes with different directions and different lengths and had varying depths in their course. According to the information given by the mother, the girl wanted to take a shower late in the evening and turned on a heating gas stove placed near the entrance of the bathroom, which gave off a distinct smell of gas, but this was not considered suspicious at the time. In the bathroom there was also an electric radiator that was switched on at the highest level and connected to electricity (Fig. 3).

According to the statements, the girl started suddenly to feel dizzy, she sat down on the toilet and then fell sideways against the radiator and slid down it. When her mother realised this, she first tried to free her daughter by pulling on both the radiator and her daughter. She then called loudly for help so that neighbours came and called the fire brigade. The mother´s partner was also found unconscious in the corridor. Both were diagnosed with initial carbon monoxide poisoning, which was subsequently treated at the hospital. The injuries, which were mainly in the form of stripes, irregular and of varying depth, were located on one side of the body and of a width compatible with the thickness of the different parts of the radiator (pipes). Also in this case, the characteristics of the lesions, with different depths and different orientations, as well as intersecting with each other and all localised on the left side of the body (therefore consistent with the girl’s fall towards the left side of the body and consequent contact with the radiator as well as ‘sliding’ on it) made the accidental event consistent, ruling out child abuse.

### Case 3

**Fig. 4:**
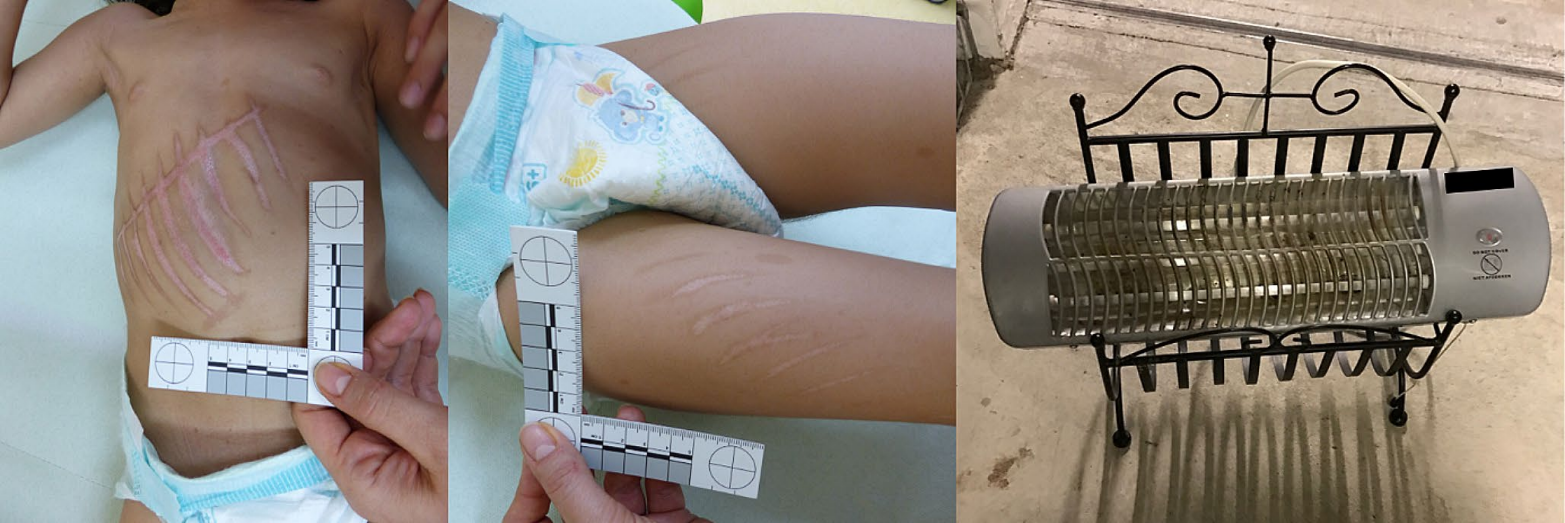
Case 3 – Burns on the chest/abdomen (left) and on the front of the thighs (middle). The portable heater responsible for the injuries placed on the stand (metal newspaper holder) as reported by the parents (right)

A 3-year-old girl was hospitalized with second-degree burns on her thorax-abdomen, displaying a well-defined shape consisting of several parallel stripes, very well defined in relation to the surrounding skin, with an almost vertical course intersected by a burn with a transverse course. Moreover, the child had been treated in hospital three months before for similar burns on her thighs, which at the examination healed into scars. The child’s parents reported that the burns in both cases resulted from the child accidentally falling on a portable heater, a type that is usually fixed to the wall but which the parents kept in the house resting on a metal magazine rack placed on the floor (Fig. 4).

The heater was stuck and secured inside the rack, with no possibility of movement or slipping. At the examination the child was generally showing good health, there was no evidence of pathology. She showed particular pain when touching the burnt parts. As to the manner of the burns, she was unable or unwilling to report what had happened. The comparative analysis of the characteristics of the injuries and those of the object (in relation to its positioning) led to the assessment that an accidental event could not have caused the type of injuries detected. Placed in that position, it was almost impossible for the child to come into contact with the heating components capable of producing the injuries at the same time. If the fall had been on the side (right or left) of the object, the lesions would have presented a completely different morphology. If, on the other hand, the fall had occurred from the front, the presence of the metal parts of the stand would have prevented all that part of the body from coming into contact with the hot parts. Furthermore, in the case of an accidental event, one would have expected less regular injuries and the involvement of a less extensive body surface. The presence of the same injuries on the thighs about three months earlier is another possible indicator of child abuse. Although by the previous burns an accidental event cannot be ruled out, as the location could be compatible with an accidental fall onto a glowing heating component, the possibility that the heater could have been pressed against the girl’s skin (or vice versa) is certainly to be taken into account. The presence of contact injuries on two different body surfaces and at two different times led to the strong suspicion of a case of child abuse, which was later confirmed by further investigations.

## Discussion

Pediatric burns are common, and the possibility of intentional burns due to abuse must be considered in the differential diagnosis so that there is a maximum chance of recognizing unintentional injuries [1–11]. In this context in so-called “contact burns” there are therefore several parameters and criteria that are part of a “step by step” assessment leading to the distinction between accidental and intentional events. A thorough multidisciplinary assessment is therefore necessary [12–18]. In these cases, accidental injuries showed characteristics of irregularity, poor definition, multiple localisation, and in both cases the evaluation of the object responsible for the burns was fundamental: in case 1, to be able to arrive at a detailed reconstruction of the events which, although accidental, had not been initially reported by the girl’s mother: in this case, an accurate reconstruction of the scene, with measurements of the spaces and simulation using a doll were of crucial importance. In case 2, the medico-legal assessment confirmed the version of an accident given by the witnesses. The third case showed abuse, the occurrence of which is not frequently observed in the forensic field, since the vast majority of cases of radiator contact burns are accidental [19–22]. The clear and well-defined characteristics of the injuries, the inconsistency of the narrative in relation to the injuries, and the detailed analysis of the object led to the correct diagnosis and discovery of child abuse. Research within forensic and pediatric domains underscores the importance of a systematic evaluation of burn injuries. This includes thorough documentation of the injury’s characteristics, photographic evidence, and, when possible, an interdisciplinary review by medical professionals, social workers, and law enforcement. Literature points to specific burn patterns, such as symmetrical burns, burns with clear demarcation lines, and injuries inconsistent with typical childhood accidents, as indicative of potential abuse. Moreover, forensic guidelines emphasize the importance of considering the social and familial contexts when assessing burn injuries for potential abuse. The analysis of the injuries is not sufficient: an inspection with measurements of the instruments involved and the reconstruction of the event is necessary, possibly with the help of doll models as in case 2, which led to a detailed reconstruction of the position of the child at the moment of contact with the radiator and thus the dynamics of the accidental event. This should be considered the gold standard in evaluating all cases of suspected child abuse and must be an indispensable cornerstone of a forensic assessment in this context.

## Data Availability

[5, 6, 7, 8, 9, 10]
